# Effects of Multidomain Interventions on Sarcopenia

**DOI:** 10.14283/jarlife.2024.9

**Published:** 2024-05-22

**Authors:** M. Nunes-Pinto, R.G. Bandeira de Mello

**Affiliations:** 1. Gerontopôle de Toulouse, Institut du Vieillissement, Centre Hospitalo-Universitaire de Toulouse, France; 2. Postgraduate Program in Medical Sciences (Endocrinology), Federal University of Rio Grande do Sul (UFRGS), Porto Alegre, Brazil; 3. Master of Public Health Program, Bloomberg School of Public Health, Johns Hopkins University, Baltimore, USA

**Keywords:** Sarcopenia, multidomain interventions, nonpharmacological treatments, muscle health, disease-modifying effects

## Abstract

Sarcopenia, a complex muscular condition driven by multi-systemic dysregulation and its interactions with lifestyle, physical attributes, and mental health, lacks effective drug treatments, relying primarily on non-pharmacological interventions. Fragmented approaches may prove suboptimal due to its complexity, underscoring the potential for multidomain interventions—a combination of two or more strategies to improve individual health—as a promising treatment option. This review examines the possible roles of multidomain interventions in sarcopenia, specifically addressing their effects on muscle mass and quality, muscle strength, and physical performance in older adults. While the updated literature highlights the beneficial consequences of multidomain interventions in enhancing physical performance outcomes, gaps persist in understanding their influence on the biological aspects of sarcopenia. Promising initial findings suggest changes in plasma inflammatory markers or muscle turnover networks, but further research is necessary to clarify the disease-modifying effects of multidomain intervention in sarcopenic patients.

## Introduction

**T**he Multidomain intervention is a broad term encompassing strategies involving at least two interventions, typically associating physical exercise, nutrition, cognitive training, or psychosocial components (1). Due to its simultaneous multiple targets, multidomain interventions are believed to promote comprehensive improvement in an individual’s health and prevent disability (2). Sarcopenia is a complex geriatric syndrome characterized by the progressive loss of muscle mass or quality, reduced strength, and declining physical performance (3). It is an age-related disease with systemic impacts, contributing to disability and mortality (4), thus making it a potential candidate for multidomain interventions. Although multidomain interventions may apply as a therapeutic option for sarcopenia, the extent to which they intervene on the pathophysiology of the disease (i.e., disease-modifying effect), on sarcopenia symptoms, or on both is unknown. The aim of this short review is to gather current data on the potential roles of non-pharmacological multidomain interventions in the treatment of sarcopenia.

## Methods and Results

A comprehensive and sensitive search strategy was conducted in the PubMed database, combining multidomain and sarcopenia terms to identify relevant evidence in this field. We looked for randomized controlled trials (RCT) that operationalized a multidomain intervention in older people. Very limited research was uncovered regarding sarcopenic populations. Considering established connections between sarcopenia and frailty (5), we included studies enrolling participants with sarcopenia, pre-frailty or frailty, and mobility impairment (low short physical performance battery (SPPB) scores).

All RCTs evaluated older adults aged 65 years or older. The interventions, lasting from 12 weeks to 3 years, always comprised physical exercise, usually along with nutritional counseling or supplementation. Only two studies did not involve any nutritional strategy (6, 7). Additional components like cognitive training, psychosocial support, or comorbidities management were sometimes aggregated. Further details about included studies are summarized in Table 1.

**Table 1.** Summarized information on studies included in the review

Study	Population	Multidomain components	Intervention and Groups	Main results
Bernabei et al.(8) (2022)	Older adults ≥70 years with sarcopenia IG: n=760 (605 SPPB 3-7, age 79.3 ±5.9, F 71.7%; 155 SPPB 8 or 9, age 78.3 ±5.7, F 72.9%) CG: n=759 (600 SPPB 3-7, age 79.2 ±5.8, F 70.8%; 159 SPPB 8 or 9, age 77.1 ±5.4, F 73.0%)	Moderate intensity physical activity training (aerobic, strength, balance) with technological support Nutritional counselling	Up to 36 mo; 1-4x/wk physical activity; Nutritional assessments and prescription of personalized dietary plan Groups: Intervention x control (lifestyle education)	SPPB increased more in IG than CG (BGD 1.0 (0.5 -1.6)^1^; p<0.001, in favor of the IG) In participants with SPPB 3-7, lower mobility disability (inability to complete the 400mt) in the IG x CG (46.8% x 52.7% respectively, HR 0.78, (0.67-0.92)^1^; p=0.005) Smaller decline in HGS and ALM in the IG x CG among women (BGD 0.9, 0.1-1.6^1^, p=0.028 and 0.49 (0.10-0.39)^1^, p<0.001, respectively)
Monti et al. (9) (2023)	Older adults ≥70 years with sarcopenia IG: n= 24, age 78.0 ± 6.1 CG: n=21, age 79.6 ± 5.8	Physical activity training (aerobic, strength, balance) Nutritional counselling	2 yrs; 1-4x/wk physical activity; Nutritional counselling Groups: Intervention x control (lifestyle education).	Better SPPB (7 (7–9)^1^to 10 (7.25–11)^1^, p=0.007) and 5CST points (1 (1–2)^1^to 2 (1.25–3)^1^, p=0.006) in IG HGS ↔ in both groups VL architecture ↔ in IG while it ↓ in CG Higher reduction in CSA in CG than IG (-12.7% vs -8.4%, respectively) CAF ↔ in IG while it ↑ in CG (+6.2%) NfL↔ in both groups Negative correlations between changes in CAF and SPPB (r = -0.305, P = 0.047)
Zhu et al.(10) (2019)	Community-dwelling older adults aged ≥65 years with sarcopenia IG: Exercise + nutrition (EN): n=36, age 74.8±6.9, 80.6% CG: Exercise alone (E) n=40, age 74.5± 7.1, 72.5% / Control (C) n=37, age 72.2±6.6, 78.4%	Physical exercise training (resistance and aerobic) Nutrient supplementation	12 wk; 2x/wk group exercise + 1x/wk home exercise session; Nutrition: High protein + β-hydroxy-β-methyl-butyrate + vitamin supplement daily Groups: EN x E x C (waitlist)	No difference in GS Better KES (+3.73 (2.28, 5.18)^1^x −0.62 (−2.17, 0.92)^1^, p<0.001) and 5CST (−3.77 (−4.76, −2.77)^1^x −1.49 (−2.55, −0.43)^1^, p=0.004) in IG x CG, respectively Better ASMI in IG x CG (+0.11 (0.03, 0.19)^1^x −0.05 (−0.14, 0.03)^1^, p=0.025, respectively)
Lu et al.(11) (2019)	Subgroup analysis of 92 participants with sarcopenia from a sample of community-dwelling prefrail/frail adults aged ≥65 years IG: n=78, age 69.76±4.31, 67.9% CG: n=14, age 71.00±6.65, 42.9%	Physical exercise training (resistance and balance) Nutritional enhancement Cognitive training	6 mo; Physical exercise: 2x/wk in person for 12 weeks + at home for 12 weeks; High protein and vitamin supplementation; 2-4x/mo cognitive training sessions Groups: Active interventions (nutrition, exercise, cognitive training and combination of those) x control (standard care). *secondary analysis	At 6 months, GS had the greatest change associated with active interventions, with 22 of 30 (73.3%) participants free of low GS, while 17 of 88 (19.3%) were free of low KES and 7 of 92 were free of low ASMI (7.6%).
Ma et al.(24) (2021)	Community-dwelling older adults aged ≥65 years with sarcopenia IG: Combined n=23, age 73.7±6.1, F 52.5% CG: Exercise only: n=11, age 76.4±7.4, F 54.5% / control: n=12, age 69.3±3.8, F 50%	Physical exercise training (resistance and aerobic) Nutrient supplementation	12 wk; 2x/wk group exercise + 1x/wk home exercise session; Nutrition: High protein + β-hydroxy-β-methyl-butyrate + vitamin supplement daily Groups: Exercise + nutrition supplement x exercise alone x waitlist control	Seven T cell-specific inflammatory genes (RASGRP1, BIN1, LEF1, ANXA6, IL-7R, LRRN3, PRKCQ) showed a significant pre-and post-difference in both IGs x CG + an interaction effect between intervention and gene expression levels on leg extension.
Tan et al.(6) (2023)	Pre-frail ambulatory adults ≥65 years. IG: Exercises n=80, age 73.39 ± 5.20, F 58.8% / Exercise + therapy: n=57, age 72.56±5.06, F 73.7% CG: n=187, age 71.69±4.99, F 48.7%	Physical exercise training (aerobic, resistance, balance) Dual-task training Cognitive stimulation therapy	6 mo; 2x/wk exercise program + dual cognitive and motor tasks; cognitive stimulation therapy Groups: Exercises, exercise + therapy, control group hHealth education)	Better GS (0.13 (0.07 – 0.18)^1^x -0.01 (-0.04 – 0.03)^1^, p<0.001), SPPB (0.65 (0.24 – 1.05)^1^x 0.13 (-0.12 – 0.38)^1^) and 5CST (-2.24 (-3.22 - -1.27)^1^x -0.27 (-0.88 – 0.35)^1^, p=0.002) in IG x CG, respectively, at 3 mo Better GS in IG x CG (0.15 (0.05 - 0.24)^1^x 0.02 (-0.04 - 0.05)^1^, respectively, p<0.001) at 12 mo Better ASMI in IG x CG at 12mo (-0.35 (-1.12 - 0.42)^1^x -2.68 (-3.06 - -2.31)^1^, respectively, p <0.001) ↓ TNFα in IG x CG (-1.74 (-3.43, -0.06)^1^, p = 0.043) at 3 mo No group difference for IL6, IL10 and GDF15
Casals et al.(12) (2023)	Community-dwelling individuals ≥ 65 years with at least one component of Fried’s frailty phenotype IG: n=80, age 73.1 (IQR 68.5–76.5), F 78.8% CG: n=83, age 74.9 (IQR 70.8–80.7), F 55.4%	Educational sessions on physical activity Healthy dietary habits counselling Cognitive training Psychological well-being and social activities	6 mo; 4 group sessions + 6 follow-up phone calls Groups: Intervention x control (usual healthcare)	Better GS (–0.4 (−0.5– −0.3)^1^x 0.5 (0.1–0.0)^1^, η2p = 0.122, p < 0.001), SPPB (1.0 (0.0–3.0)^1^x −0.1 (−1.2–1.0)^1^, η2p = 0.179, p < 0.001) and 5CST (−4.3 (−7.0– −2.3)^1^x 1.0 (0.0–1.2)^1^, η2p = 0.136, p < 0.001) in IG x CG, respectively Lower prevalence of low HGS in the IG (pre-post-intervention: 48.8% x 25% the IG; 83.1% x 83.1% in the CG, p < 0.001)
Cameron et al.(13) (2013)	Rehabilitation outpatients ≥70 years with ≥3 CHS frailty criteria IG: n=120, age 83.4 ±5.81, F 67% CG: n=121, age 83.2 ±5.91, F 68%	Individualized exercise program (balance and strength) Management of medical, nutritional, and psychosociological problems	12 mo; 10 physiotherapy sessions + home exercises prescribed; High energy, high protein supplements. Groups: Intervention x control (usual care)	SPPB stable in the IG while it decreased in CG (IG +0.52 (±2.47) x CG -0.98 (±2.30), BGD 1.44 points (0.80, 2.07)^1^, p < 0.001)
Fairhall et al. (14) (2014)	Rehabilitation outpatients ≥70 years with ≥3 CHS frailty criteria IG: n=120, age 83.4 ±5.81, F 67% CG: n=121, age 83.2 ±5.91, F 68%	Individualized exercise program (balance and strength) Interdisciplinary management of medical, nutritional, social, and psychological problems	12 mo; 10 physiotherapy sessions + home exercises prescribed; High energy, high protein supplements. Groups: Intervention x control (usual care)	Better SPPB and GS in the IG (BGD 1.58, (1.02-2.14)^1^, p≤ 0.001 and 0.06 (0.01-0.10)^1^, p= 0.02, respectively, in favor of IG)
Gené Huguet et al. (16) (2018)	Community-dwelling pre-frail adults aged ≥ 80 years. IG: n=100, age 84.5 ±3.4, F 68% CG: n=100, age 84.5 ±3.7, F 61%	Physical exercise training (aerobic, strength, resistance, balance) Mediterranean diet advice, inadequate prescribing review, and social assessment	6 mo; 9 exercise sessions + home exercise prescription (3-4x/wk), 1 nutrition session, assessment of polypharmacy, personal and social support by telephone calls. Groups: Intervention x control (usual primary care)	Better TUG (BGD -1.57 (-2.36, -0.78)^1^, p<0.001) and 5CST (BGD -2.46 (-3.87, -1.06)^1^, p=0,001) in the IG x CG Prevalence of slow GS stable in IG (11.8% to 12.9%, p=1), increased in CG (10.2% to 22.7%, p=0.01)
Kim et al.(17) (2015)	Frail women ≥75 years IG: Ex + MFGM; n=33, age 81.0±2.6, F 100% CG: Ex + Placebo n=33, age 81.1±2.8 / MFGM n=32, age 81.0±2.8 / placebo n=32, age 80.3±3.3; F 100%	Physical training program (balance and resistance) Milk fat globule membrane (MFGM) supplementation	12 wks; 2x/wk exercise class. Milk fat globule membrane (MFGM) supplement every two weeks. Groups: Exercise + MFGM (multidomain) x exercise + placebo x MFGM x placebo	Better GS (+14.7% (6.4 - 23.1)^1^x +3.6% (1.9, 9.1)^1^, p=0.026) and TUG (-14.1% (-13.8,-9.9)^1^x -3.0% (-8.3, 2.3)^1^, p<0.001) in the multidomain IG x CG, respectively No difference in ALM, HGS or KES ↑ BDNF (6.60±1.54 to 7.18±1.09) and ↓ myostatin (54.48±14.92 to 45.75±16.02) in the multidomain IG (within group) ↓ IGFBP-3/IGF-1 in the multidomain IG, while it ↑ CG (5.50±2.28 to 5.02±1.96 x 4.65 1.72 5.38 1.93, p=0.013 No differences in B2 microglobulin and GH levels
Kwon et al. (18) (2015)	Prefrail women aged ≥70 years from the community. IG: EN n=26, age 76.5±3.8, F 100% CG: E n=25, age 77.0±4.2 / C n=28, age 76.9±3.9; F 100%	Physical exercise training (strength and balance) Nutritional program	12 wks; 1x/wk physical exercises; 1x/wk nutritional advice and healthy cooking class. Groups: Exercise + nutrition (EN), exercise only (E), control (C)(health education)	No difference in GS and HGS
Ng T et al.(19) (2015)	Community-dwelling pre-frail and frail individuals aged ≥65 years IG: Combined n=49, age 70.4 ±4.74, F 53.1% CG: Nutritional n=49, 69.7 ±4.2, F 66% / Cognitive: n=50, 69.7±4.3, F 76% / Physical: n=48, age 70.3 ±5.2, F 56.2% / Control: n=50, age 70.1 ±5.02, F 56%	Physical exercise training (strength and balance) Nutritional counseling Cognitive training	6 mo; Physical exercise: 2x/wk in person for 12 wks + at home for 12 wks; Daily high protein and vitamin supplementation; 2-4x/mo cognitive training sessions Groups: Nutrition x cognitive x physical training x multidomain (combination of the previous three) x control (usual care)	No difference in GS Better KES in the multidomain IG x CG (2.35 (1.25-3.44)^1^x -0.24 (-1.34-0.87)^1^, time*group p=0.009)
Rydwik et al.(20) (2008)	Community-dwelling prefrail adults aged ≥75 years IG: T+N n=25, 83.1 ±4, F 64% CG: N n=25, age 83.1± 4.5, F 60%; T n=23, age 83.5 ±3.7, F 47.8%, C n=23, age 82.9 ±4, F 69.5%	Physical training (strength, balance, functional) Nutritional intervention program	12 wks; 2x/wk multicomponent physical exercise. Nutritional treatment based on diet records. Groups: Training + nutrition (T+N) x nutrition N x Training T x control (general health advice)	No difference in TUG Better leg press strength (+9kg (1.8, 16.2)^1^x 2.4kg (-2.9, 7.7)^1^, p<0.05) and CST (1.2 (0.07, 2.3)^1^x -0.2 (-0.9, 0.6)^1^, p<0.05) in IG x CG, respectively
Caldo-Silva et al.(21) (2021)	Frail/prefrail long-term-care-facility residents ≥70 years IG: E + BCAA n=8 age 80 ± 6.1 CG: ME n = 7, age 86.7 ± 4 / BCAA n = 7, age 84.2 ± 5.8 / CG n = 13, age 83.1 ± 5.4. Data on sex not shown.	Exercise training (aerobic, strength, resistance, balance) Nutrient supplementation (BCAA)	16 wks / 8wks washout / 16 wks; 2/week exercise training. BCAAs power mixture daily. Groups: Exercise x Exercise + BCAA (multidomain) x BCAA x control	Better 5CST in the multidomain IG (within group, 21.87 (±3.64) to 18.71 (±3.59), p = 0.009) Decrease of TNF-α levels in a second wave of 16 weeks of intervention (after a washout detraining period) in multidomain IG (within group 112.86 (±62.51) to 57.37 (±31.18), p<0.01) No difference in IL-10, MPO and albumin.
Caldo-Silva et al.(25) (2023)	Frail/prefrail long-term-care-facility residents ≥70 years IG: E+BCAA n=8 age 80 ± 6.1 CG: ME n = 7, age 86.7 ± 4 / BCAA n = 7, age 84.2 ± 5.8 / CG n = 13, age 83.1 ± 5.4. F% not shown.	Exercise training (aerobic, strength, resistance, balance) Nutrient supplementation (BCAA)	16 wks / 8wks washout / 16 wks; 2x/wk exercise training. BCAAs power mixture daily. Groups: Exercise x Exercise + BCAA, BCAA, c,ontrol	No differences in the hematological profile (hemoglobin, erythrocytes, white blood cells or platelets counts).
Stathi et al.(7) (2022)	Older adults (aged ≥65 years) with mobility impairment (SPPB 4–9) IG: n=410, age 77.8 ± 6.93, F 67% CG: n=367, age 77.3 ± 6.64, F 66%	Multimodal physical activity (strength and balance) Behavioral change technique (focused on psycho-sociological aspects)	12 mo; 1-2x/wk physical exercise. 1x/wk social activity and behavioral maintenance program Groups: Intervention x control (health education)	Better SPPB in IG x CG (8.08±2.87 x 7.59±2.61, respectively; mean difference 0.49 (0.06–0.92)^1^, p=0·014) No difference in HGS
Skoglund et al.(15) (2022)	Mobility-impaired (SPPB ≤9) and Vitamin D insufficient (9-24 ng/mL) older adults over 70 years. IG: n=23, age 77.4± 5.6. CG: n=26, age 77.1 ±5.4 Data on sex not shown	Physical activity program (flexibility, balance, strength, aerobic) Nutritional protein-rich and vitamin D supplement	6 mo; 3x/wk physical activity sessions; 1/Daily nutritional supplement (20 g of whey protein, 800 IU of vitamin D, and other vitamins and minerals) Groups: Exercise + supplement x exercise (control)	↑ CSA in the knee extensors (+1.9%, p < 0.01) and the hip adductors (+2.8%, p < 0.05) in both groups, with or without nutritional supplement ↓ RA in the hip flexors (1.1, p< 0.01), hip adductors (0.9HU, p < 0.05), knee extensors (1.2, p< 0.001) and ankle dorsiflexors (0.8, p <0.05) While GS and SPPB improved, multivariate analysis showed that these changes were not associated with the changes in muscle CSA and RA after the intervention.
Englund et al.(22) (2018)	Mobility-impaired (SPPB ≤9) and Vitamin D insufficient (9-24 ng/mL) older adults over 70 years IG: n=74, age 78.1 ±5.8, F 46% CG: n=76, age 76.9 ±4.9, F 47%	Physical activity program (flexibility, balance, strength, aerobic) Nutritional protein-rich and vitamin D supplement	6 mo; 3x/wk physical activity sessions; Daily nutritional supplement (20 g of whey protein, 800 IU of vitamin D, and other vitamins and minerals) Groups: Exercise + supplement x exercise (control)	Better thigh composition (higher thigh muscle CSA (+1.51 (0.59, 2.43)^1^and +3.03 (1.69, 4.38)^1^), lower subcutaneous adipose tissue (−2.2 (−3.71, −0.69)^1^and −2.57 (−4.91, −0.24)^1^) and lower intermuscular fat (−0.26 (−0.45, −0.07)^1^and −0.63 (−0.93, −0.32)^1^) in both groups (exercise without or with supplementation, respectively) Nutritional supplementation led to further losses of intermuscular fat (−12% x −5%, p=0.049) and increased normal muscle density (+6% x +1%, p=0.018) No difference in ALM after interventions

Legend: IG Intervention group; CG Control group; n Number of participants; F female participants; mo Month; wk Week; x/ times per; HR Hazard ratio; ^1^95% confidence interval; BGD Between-group difference; HGS Handgrip strength (kilogram); GS gait speed (meter per seconds); SPPB Short physical performance battery scores (points); 5CST Five chair stand test (seconds); TUG Timed Up and Go test (seconds); 400mt 400m walk test; KES Knee extension test (kilograms); ALM Appendicular lean mass (kilograms); ASMI Appendicular skeletal muscle mass index (ASM/height^2^. Kilograms per meters^2^); CHS Cardiovascular Health Study; FNIH Foundation for the National Institutes of Health; PUFA Polyunsaturated fatty acid; IADL Instrumental Activities of Daily Living; IU International units; TNFα Tumor necrosis factor alpha (pg/mL); CSA Cross sectional area (cm^2^), RA radiological attenuation (Hounsfield units). Please refer to the original articles for further information.

The collected data refers to the effects of multidomain interventions in each of the components of sarcopenia (muscle mass/quality, muscle strength, or physical performance). For organizational clarity, the findings are here categorized into these three components in alignment with the consensus of the European Working Group on Sarcopenia in Older People (3). Finally, information related to the biological influences of multidomain interventions (disease-modifying effects) will be presented.

### Physical performance

Regarding multidomain interventions in participants diagnosed with sarcopenia, two studies identified greater improvement in the SPPB total score in the multidomain intervention group compared to control group (8, 9). While one study found no differences between groups in gait speed (GS) after 12 weeks of intervention (10), Lu et al. identified that among the components of sarcopenia, GS exhibited the greatest change associated with multidomain intervention, with 22 out of 30 participants becoming free of low GS after 6 months (11). Moreover, in a multicenter European study involving 1519 older adults, a subgroup analysis of participants with lower SPPB score (3 to 7) revealed that the multidomain intervention was associated with a reduced risk of mobility disability (inability to perform the 400-meter walk test) during the 36-month follow-up (8).

Similar findings were seen among pre-frail/frail or mobility-impaired samples. Several studies identified greater improvement in the SPPB total score (6, 7, 12–15), GS (6, 12, 14–17) and timed-up-and-go test (TUG) (16, 17) in the multidomain intervention group compared to control groups. Only three studies found no difference between groups in GS (18, 19) and TUG (20).

### Muscle strength

Examining samples with diagnosed sarcopenia, three studies evaluated muscle strength. Two of them found improved completion time for the 5-times chair sit-to-stand test (5CST) after the intervention compared to the control group (9, 10). Additionally, knee extension strength (KES) was greater in the intervention group than in the control group (10). While one study found no difference between groups in handgrip strength (HGS) after a two-year intervention (9), Bernabei et al. identified a smaller decline in HGS over the same period in women assigned to the multidomain intervention than those in the control group; however, no difference was seen among men (8).

In the two other populations, several studies also identified improvements in the 5CST (6, 12, 16, 20, 21) and lower limb strength (19, 20, 22) with the intervention compared to the control group, except for one study that did not find differences in KES between the groups (17). Casals et al. found a decrease in the prevalence of individuals with low HGS in the intervention group, with no significant changes observed in the control group (12). However, three other studies found no significant difference in HGS between groups (7, 17, 18).

### Muscle mass and quality

Regarding individuals with sarcopenia, Zhu et al. identified increased appendicular skeletal muscle mass (ASM) index (ASM/height^2^) in the combined exercise-program-and-nutrition-supplement group over 12 weeks, while it decreased among controls (10). Bernabei et al. also found that women that received the multidomain intervention experienced less loss of appendicular lean mass (ALM) compared to the control group at 36 months. However, no significant differences were observed in men (8). Interestingly, in a sample of 92 sarcopenic individuals, among the three components of sarcopenia, muscle mass exhibited the least change, with only 7.6% achieving a normal ALM index (ALM/height^2^) after 6 months (11). In the other populations, one study observed a smaller decline in ASM index among the intervention group compared to controls. (6). Two other studies found no group differences concerning ALM (17, 22), although in one of them, the control group also received physical exercise (22).

More recently, studies have analyzed muscle quality parameters using image assessment techniques such as computed tomography scans (CT) or ultrasound (US). Performing US measurements on the vastus lateralis muscle of sarcopenic individuals, Monti et al. demonstrated preserved muscle architecture (pennation angle and fascicle length) despite a reduction in cross-sectional area (CSA) after the intervention. Conversely, in the control group, these architectural parameters declined along with a further decrease in CSA (9). Two studies assessed thigh measurements in mobility-impaired individuals. Within the intervention group, Englund et al. verified CT improvements in thigh composition (higher thigh muscle CSA, lower subcutaneous adipose tissue, lower intermuscular fat) (22). Skoglund et al. also observed significant increases in CT CSA and enhanced radiological attenuation, a parameter of muscle density, in knee extensors and hip adductors following the intervention. However, despite improvements in physical performance within this sample, multivariate analysis revealed that these changes were not directly associated with the alterations in muscle CSA and density post-intervention (15).

### Biological evaluations

Ongoing research into the pathophysiology of sarcopenia reveals a complex multisystemic dysregulation indicative of altered muscle anabolic and catabolic responses, chronic inflammation, mitochondrial dysfunction, and neuromuscular changes (23). Two of the included studies assessed biological parameters after multidomain interventions in sarcopenic individuals. Through the analysis of blood T cells, Ma et al. discovered significant differences in the expression of seven genes associated with T cell regulation and inflammation (RASGRP1, BIN1, LEF1, ANXA6, IL-7R, LRRN3, and PRKCQ) between pre- and post-intervention measurements. These differences were found to correlate with leg extension strength (24). Moreover, Monti et al. found that C-terminal agrin fragment (CAF) levels, a marker of neuro-muscular junction (NMJ) instability, remained unchanged in the intervention group. At the same time, they increased in the control group after a two-year follow-up. They also observed a correlation between better SPPB scores and lower CAF concentration. Conversely, no difference was found in plasma levels of neurofilament light chain, another marker of the motoneuron health (9).

Other authors assessed biomarkers following multidomain interventions in pre-frail/frail populations. Concerning inflammation, Caldo-Silva et al. verified that plasma tumor necrosis factor alpha (TNF-α) levels decreased in the group receiving physical exercise and branched-chain amino acids supplementation, but only after a detraining period followed by a second wave of 16 weeks of intervention (21). Tan et al. also identified a reduction of TNF-α levels after 12 weeks of intervention compared to controls (6). No differences were seen among groups for other inflammatory indicators such as interleukin (IL) 6 (6), IL-10 (6, 21), and myeloperoxidase (21), nor in the hematological profile (hemoglobin, erythrocyte, white blood cell, or platelet counts) (25). Markers related to nutrient sensing and muscle turnover were also assessed. One study verified lower insulin-like growth factor (IGF) Binding Protein-3 to IGF-1 plasma ratios in the multidomain group compared to placebo, alongside reduced myostatin and increased brain-derived neurotrophic factor serum levels within the group (17). After the intervention, no difference was seen concerning growth differentiation factor 15 (6), Beta-2 microglobulin (17), albumin (21), and growth hormone (17).

### Final considerations and future perspectives

Sarcopenia is a multifaceted geriatric syndrome with significant implications for both individuals and society, as it is associated with quality of life, independence, morbidity, and mortality (3). As of today, there is currently no evidence supporting drug treatments for sarcopenia; strength exercise training and nutritional support remain the main management options (26). Therefore, multidomain interventions are an interesting approach, especially when combining these last two aspects.

**Figure 1:**
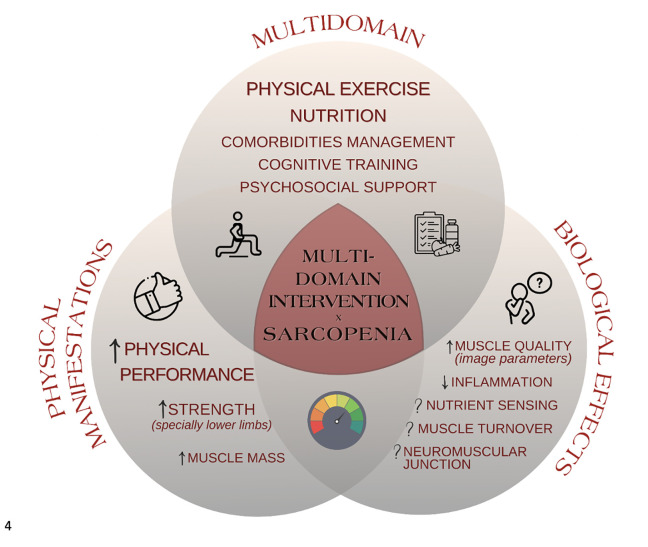
A summarized framework of multidomain interventions on sarcopenia and its components, including modifications in physical manifestations and potential biological effects, according to the main findings of our review

Findings from this review, summarized in figure 1, indicate beneficial impacts of multidomain interventions on the muscular components of sarcopenia among older adults. Muscle performance appears to be the more consistently improved aspect. Greater lower limb strength measured by CST or KES was also observed. Muscle mass seems to ameliorate with multidomain intervention, probably in a smaller degree compared to the other components. Conversely, the variability in improvements in HGS suggests a less uniform response. This could arise from several factors, including larger muscle mass volume, better response to interventions (27), or more pronounced age-related declines in lower limb muscle compared to the upper body (28). Moreover, lower limbs are extensively involved in weight-bearing and functional activities, and balance and mobility exercises often prioritize the lower body (29). Overall, responses seemed to be influenced by age, sex, degree of baseline impairment, and the type of intervention. Although not within this review’s scope, it’s important to recognize that since all studies combined physical exercise with another modality, and typically compared the intervention to a control group, the exclusive effect of physical exercise cannot be ruled out.

While multidomain interventions demonstrate a more consistent impact on the physical manifestations of sarcopenia, there is still limited understanding of their pathophysiology effects. Recent research delves into qualitative changes in the muscle, with modifications in architectural parameters and intramuscular fat being seen even without major changes in muscle mass. Furthermore, there may be primary effects concerning some inflammatory markers, nutrient sensing, and muscle turnover networks, or measurements of the neuromuscular junction. Still, current data is derived from a limited number of studies with small sample sizes, often lacking comprehensive analysis of biological parameters and their correlation with physical outcomes. Future research addressing larger populations aimed specifically at sarcopenic patients is needed, with a special focus on the biological primary pathways and disease-modifying effects of multidomain interventions.
